# The Ratio of C-Reactive Protein/Albumin is a Novel Inflammatory Predictor of Overall Survival in Cisplatin-Based Treated Patients with Metastatic Nasopharyngeal Carcinoma

**DOI:** 10.1155/2017/6570808

**Published:** 2017-06-06

**Authors:** Peng Sun, Cui Chen, Yi Xia, Xiwen Bi, Panpan Liu, Fei Zhang, Hang Yang, Xin An, Wenqi Jiang, Fenghua Wang

**Affiliations:** ^1^State Key Laboratory of Oncology in South China, Collaborative Innovation Center for Cancer Medicine, 651 Dong Feng RD East, Guangzhou, Guangdong Province 510060, China; ^2^Department of Medical Oncology, Sun Yat-Sen University Cancer Center, 651 Dong Feng RD East, Guangzhou, Guangdong Province 510060, China; ^3^Department of Oncology, The First Affiliated Hospital of Sun Yat-Sen University, 58 Zhongshan Road II, Guangzhou, Guangdong Province 510080, China

## Abstract

The C-reactive protein/albumin (CRP/Alb) ratio has been recently identified as a prognostic factor in various cancers, whereas its role remains unclear in metastatic nasopharyngeal carcinoma (NPC). The current study retrospectively analyzed 148 patients with metastatic NPC who underwent cisplatin-based chemotherapy and further evaluated the prognostic value of the CRP/Alb ratio and its association with clinical characteristics in these patients. The optimal cut-off value was 0.189 for the CRP/Alb ratio. The high CRP/Alb ratio was significantly associated with elevated NLR, platelet-to-lymphocyte ratio (PLR), and EBV-DNA levels and decreased haemoglobin level (all *p* < 0.05). The results of multivariate analysis showed that the CRP/Alb ratio was an independent prognostic factor of overall survival. Patients with a high CRP/Alb ratio (≥0.189) had a 1.867 times (*p* = 0.024, 95% CI = 1.085–3.210) greater risk of mortality compared with those with a low CRP/Alb ratio (<0.189). In addition, combining the CRP/Alb ratio with GPS could accurately discriminate the prognosis of our patients. Our results suggested that the CRP/Alb ratio is a feasible and inexpensive tool for predicting survival outcomes and is a valuable coadjutant for the GPS to further identify differences in survivals of patients with metastatic NPC.

## 1. Introduction

Nasopharyngeal carcinoma (NPC) is a distinct head and neck cancer with unique etiological and epidemiological feathers, with the incidence varying from 0.5–3/100,000 per year in North Africa to 20–30/100,000 in certain epidemic areas, such as South China and Southeast Asia [1–4]. Radiotherapy is the primary treatment of NPC, and the development of intensity-modulated radiotherapy technology (IMRT) and new chemotherapy agents significantly improves the cure rate [5–7]. However, more than 20% of NPC patients will ultimately develop distant metastasis after definitive chemoradiotherapy [8]. On the other hand, 5%–6% of NPC patients have disseminated disease at the time of initial diagnosis [8]. Hence, metastatic disease remains a critical problem and a leading cause of death among patients with NPC. For patients with metastatic NPC, systematic chemotherapy is the standard therapeutic option and has been well established [9]. Although multiple agents and regimens have been explored, the palliative chemotherapy for NPC has rarely been studied in any randomized trials yet. Cisplatin-based multiple chemotherapy was reported to reach a response rate of 50–80% in NPC patients; it has been recommended as the standard first-line regimen for metastatic NPC [10–12]. However, the clinical outcome varied significantly in patients with metastatic NPC. Therefore, it is critically important to find predictive factors for prognosis, which could help us to accurately select the patients who respond well to cisplatin-based chemotherapy.

Since the well-known tumor-node-metastasis (TNM) staging system could not provide prognostic information, a series of clinical characteristics and laboratory biomarkers associated with prognosis of patients with metastatic NPC have been explored in several studies [13–19]. Hemoglobin, performance status (PS), and disease-free interval (DFI) were first identified as prognostic factors for disseminated NPC by Toh et al. [20] in 2005. Then, lactate dehydrogenase (LDH) was also found to be significantly associated with survival of patients with metastatic NPC [21, 22]. Epstein-Barr virus (EBV) was a steady prognostic factor and predictor of treatment response for patients with metastatic NPC who underwent palliative chemotherapy [21, 23, 24]. In 2012, Jin et al. [21] built a prognostic model including EBV-DNA load, PS, LDH, alkaline phosphatase (ALP), and hemoglobin and successfully stratified the patients into different risk groups.

The role of systematic inflammatory response has been increasingly appreciated in multiple cancers; inflammatory biomarkers, such as C-reactive protein (CRP) level [25], Glasgow Prognostic Score (GPS) [26], modified Glasgow Prognostic Score (mGPS) [27], neutropil to lymphocyte ratio (NLR) [26], and monocyte to lymphocyte ratio (MLR) [28, 29], have already showed prognostic value in metastatic NPC. Xia et al. [25] retrospectively analyzed 116 patients with metastatic NPC who underwent palliative chemotherapy and found that baseline CRP level was significantly associated with survival. In 2014, Chen et al. [26] reviewed 211 Chinese patients with disseminated NPC who all received cisplatin-based chemotherapy and further validated the prognostic value of GPS and NLR. Recently, the CRP/albumin (CRP/Alb) ratio was recognized as a novel inflammatory index in infectious disease [30, 31]. In addition, the CRP/Alb ratio has showed promising prognostic value in gastric cancer [32], esophageal cancer [33], colorectal cancer [34], small cell lung cancer [35], and even in nonmetastatic NPC [36].

However, to the best of our knowledge, there is no data regarding the prognostic value of the CRP/Alb ratio in the metastatic NPC. Therefore, we conducted this study and aimed to examine the prognostic value of the CRP/Alb ratio and its association with clinical characteristics in patients with disseminated NPC.

## 2. Patients and Methods

### 2.1. Ethics Approval and Consent to Participate

All the patients offered informed consent for collection of clinical characteristics and pathologic information. The study was approved by the Bioethics Committee of the Sun Yat-Sen University Cancer Center for a retrospective analysis of the collected data. The study was undertaken in accordance with the ethical standards of the World Medical Association's Declaration of Helsinki.

### 2.2. Patients

We retrospectively analyzed the records of patients with NPC who were treated at the Sun Yat-Sen University Cancer Center between January 2008 and October 2011. All the patients met the following criteria: (a) the disease was pathologically diagnosed as NPC and evaluated clinically to be metastatic disease; (b) complete pretreatment data of CRP and albumin were available; (c) at least two cycles of first-line cisplatin-based palliative chemotherapy were administrated; (d) Karnofsky Performance Scores (KPS) were ≥60; and (e) hepatic and renal functions were normal. Exclusion criteria were as follows: (a) patients with brain metastases; (b) patients with other types of malignancy; or (c) patients with clinical evidence of infection or other inflammatory disease. Finally, a total of 148 patients were identified and originated the current study cohort.

### 2.3. Treatment and Follow-Up

The following three cisplatin-based chemotherapy regimens were administrated every 21 days:

- TP regimen: paclitaxel (175 mg/m^2^ intravenously (IV) over 3 hours with standard premedication on day 1 of a 21-day cycle) plus cisplatin (25 mg/m^2^ IV on days 1–3 of a 21-day cycle)
- PF regimen: cisplatin (25 mg/m^2^ IV on days 1–3 of a 21-day cycle) plus 5-fluorouracil (500 mg/m^2^ IV on days 1–5 of a 21-day cycle)
- TPF regimen: paclitaxel (135 mg/m^2^ IV over 3 hours with standard premedication on day 1 of a 21-day cycle) plus cisplatin (25 mg/m^2^ IV on days 1–3 of a 21-day cycle) plus 5-fluorouracil (800 mg/m^2^, continuous IV infusion for 24 hours, on days 1–5 of a 21-day cycle).

After treatment, all patients were followed up every 3 months. Physical and radiological examinations were performed every 3 months or when clinical progression of disease was indicated. The last follow-up was performed on December 31, 2013. Overall survival (OS) was defined as the time interval from the initial diagnosis of metastasis to the date of death or the last follow-up.

### 2.4. Evaluation

Basic demographics (gender, age), baseline clinical characteristics (KPS, site of metastasis), and relevant laboratory data were collected. Hematological parameters, biochemical markers, and EBV-DNA load were examined within one week before chemotherapy. Smoking status was recorded as ever or never for all patients. Smoker ever was defined as ≥1 lifetime pack-year. The GPS, NLR, and platelet-to-lymphocyte ratio (PLR) were calculated. We calculated the CRP/Alb ratio as dividing the CRP level (mg/L) by the serum albumin level (g/L). The Response Evaluation Criteria in Solid Tumors (RECISTs) 1.0 was used to evaluate tumor response.

### 2.5. Statistical Analyses

Continuous variables, such as LDH and NLR, are presented as median value and range and were compared by using *t*-test or nonparametric test. Categorical variables, such as gender and GPS, were described as the numbers and percentages and were compared by using the chi-square or Fisher's exact test.

A web-based system, R software-engineered, designed by Budczies J et al. [37], was used to identify the optimal cutoff value of potential prognostic parameters (http://molpath.charite.de/cutoff/). The Kaplan-Meier method was used to estimate the OS. Univariate and multivariate survival analyses were performed based on the Cox proportional hazards regression methodology. Hazard ratios (HRs) with 95% CIs and two-sided *p* values were reported. An alpha value of *p* < 0.05 was considered statistically significant. The statistical analyses were performed using the Statistical Package for the Social Sciences version 19.0 (IBM, Armonk, NY, USA).

## 3. Results

### 3.1. Patient Characteristics

A total of 148 patients with metastatic NPC were included in our study. Of these, 124 (83.8%) were males and 24 (16.2%) were females; 68 (45.9%) were smokers and 80 (54.1%) were nonsmokers. The median age at initial diagnosis of metastatic NPC was 45 years (ranging 24–72 years). More than half (*n* = 79, 53.4%) of the patients had more than one metastasis site, and few of the patients (*n* = 43, 29.1%) developed synchronous metastasis. Liver metastasis, lung metastasis, and bone metastases were found in 56 patients (37.8%), 69 patients (46.6%), and 61 patients (41.2%), respectively. The baseline plasma EBV-DNA load ranged from 0 to 9.13 × 10^7^ copies/mL, with a median value of 4.82 × 10^4^ copies/mL. GPS was evaluated as 0 in 88 patients (59.5%), 1 in 46 patients (31.1%), and 2 in 14 patients (9.4%). The median values of the CRP/Alb, NLR, and PLR were 0.164, 3.3, and 181, respectively. Of the 148 patients, 12 (8.1%) were treated with the TP regimen, 45 (30.4%) with the PF regimen, and 91 (61.5%) with the TPF regimen (Table 1).

### 3.2. Survival

Using the Cutoff Finder, we determined 0.189 as the optimal cutoff value of the CRP/Alb ratio for OS. All participants were then divided into the high CRP/Alb ratio group (≥0.189, *n* = 70) and low CRP/Alb ratio group (<0.189, *n* = 78). Accordingly, binarization of other continuous data (LDH, NLR) by optimal cutoff value was performed for the subsequent analysis of OS (Table 2).

**Figure 1:**
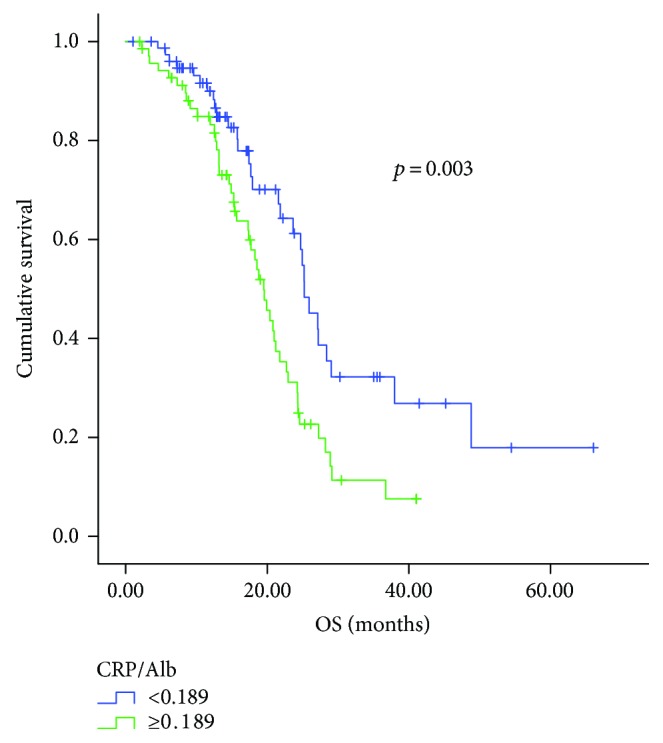
Kaplan-Meier curves for overall survival (OS) according to the CRP/Alb ratio.

Median follow-up time was 15.3 months (ranging 1 month–66 months). Of the 148 patients, 77 (52.0%) died before the last follow-up. Median OS time for the entire patient group were 21.8 months, with the 1- and 2-year OS rates of 69.6% and 22.3%, respectively. Compared with the patients in the high CRP/Alb ratio group, patients in the low CRP/Alb ratio group had significantly longer overall survival (25.2 months versus 19.5 months, *p* = 0.003) (Figure 1).

Besides the CRP/Alb ratio, GPS (*p* < 0.001), serum LDH level (*p* = 0.006), plasma EBV-DNA load (*p* < 0.001), pretreatment NLR (*p* = 0.017), PLR (*p* = 0.002), and treatment response (*p* = 0.005) were associated with OS in the univariate analysis. After adjusting for other covariates in multivariate analysis, the CRP/Alb ratio (*p* = 0.024) was proved to be an independent prognostic factor of OS, as well as GPS (*p* = 0.001) and EBV-DNA load (*p* = 0.041). We found that patients in the high CRP/Alb ratio group had a 1.867 times (*p* = 0.024, 95% CI = 1.085–3.210) greater risk of mortality compared with those in the low CRP/Alb ratio group (Table 2).

### 3.3. Association of the CRP/Alb Ratio with Clinicopathologic Characteristics

Patient characteristics were compared between subgroups by the CRP/Alb ratio (Table 1). It was found that the high CRP/Alb ratio showed a trend to correlate with multiple metastasis (*p* = 0.071) and elevated LDH level (*p* = 0.069), with marginal statistical significance. Of note, the high CRP/Alb ratio was significantly associated with high NLR, PLR, and EBV-DNA level and low haemoglobin level (all *p* < 0.05).

### 3.4. Generation of a New Prognostic Score

**Figure 2:**
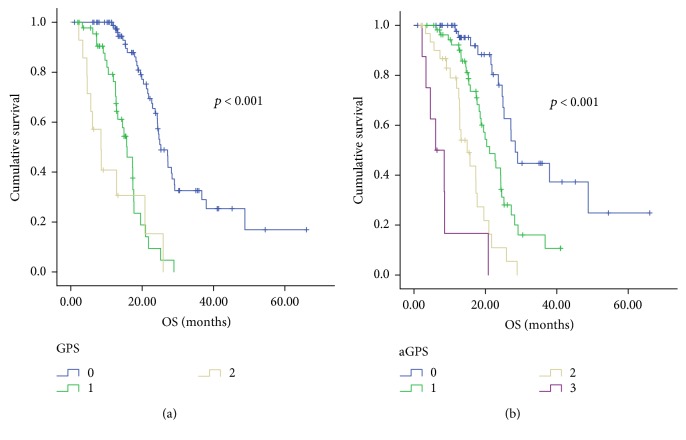
Kaplan-Meier curves for overall survival (OS) according to the GPS (a) and to the aGPS (b).

**Figure 3:**
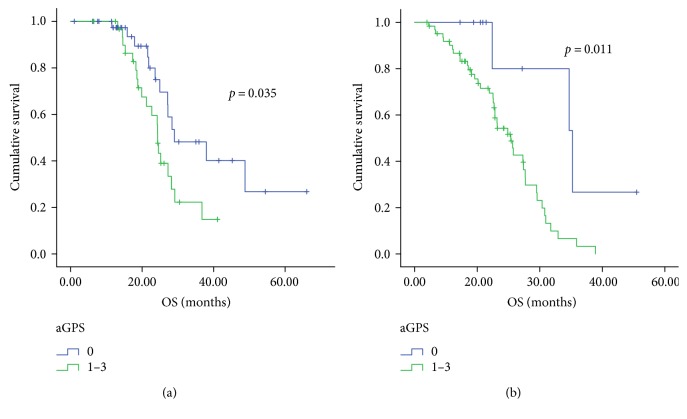
Kaplan-Meier curves for overall survival (OS) according to the aGPS in patients with pretreatment EBV DNA <4.82 × 10^4^ copies/mL (a) and in those with pretreatment EBV DNA ≥4.82 × 10^4^ copies/mL (b).

Although GPS was an independent prognostic factor in the Cox model, the difference in OS between patients with GPS 1 and those with GPS 2 was not statistically significant (15.8 months versus 8.5 months, *p* = 0.101, Figure 2(a)). Thus, we further added the CRP/Alb ratio to the GPS system and established a new prognostic score called aGPS. A score of 1 was assigned to CRP >10 mg/L, albumin <35 g/L, and the CRP/Alb ratio ≥ 0.189. Thus, of the 148 patients, 52 (35.1%) were evaluated as having an aGPS of 0, 56 (37.8%) had an aGPS of 1, 32 (21.6%) had an aGPS of 2, and 8 (5.4%) had an aGPS of 3; the median OS of these patients were 28.4 months, 21.2 months, 14.9 months, and 6.1 months, respectively (*p* < 0.001, Figure 2(b)). The OS curves of these subgroups were statistically separated from each other. Furthermore, aGPS (aGPS =0 versus aGPS >0) was also significantly associated with prognosis of NPC patients when stratified by plasma EBV-DNA level, whereas plasma EBV-DNA level (<4.82 × 10^4^ copies/mL versus ≥4.82 × 10^4^copies/mL) was not (Figure 3). Patients with aGPS 0 had longer OS than patients with aGPS >0 either in the high plasma EBV-DNA level group or in the low plasma EBV-DNA level group.

## 4. Discussion

Systematic inflammatory response has been well studied in the carcinogenesis and progression of malignancies during the past decade, and various inflammatory biomarkers have shown to be associated with cancer prognosis [38]. Serum CRP is an acute-phase protein produced by hepatocytes; it has been long recognized as a prognostic factor for many patients with malignancies including NPC [25, 39–42]. Meanwhile, hypoalbuminemia could predominantly reflect malnutrition as well as inflammatory condition and was found to be associated with impaired survival outcome of cancer patients [43]. GPS and mGPS were thus generalized from the combination of CRP and albumin; as new inflammatory markers, they have been proved to be useful prognostic factors to predict the risk of cancer.

More recently, a novel combination model of CRP and albumin—CRP/Alb ratio—has also been increasingly appreciated since it could serve as a predictor of a clinical outcome in patients with serious infectious disease and those with cancers [31, 32, 34, 35, 44]. He et al. [36] investigated the CRP/Alb ratio in nonmetastatic NPC. They found that the CRP/Alb ratio was a steady prognostic factor for the prognosis of patients with nonmetastatic NPC, and it could identify survival differences even if stratified by EBV-DNA level. However, most of previous studies on the CRP/Alb ratio limited the cohort to patients who were diagnosed with localized cancers and underwent curative therapy [32, 34, 44]. By contrast, our study focused on the patients with metastatic NPC and firstly demonstrated that the CRP/Alb ratio was an independent prognostic factor of overall survival.

Of note, the CRP/Alb ratio reflects a comprehensive state of both CRP and albumin rather than a simple addition of CRP and albumin. In contrast to GPS, the CRP/Alb ratio might indicate more comprehensive information, reflect dynamic change of systematic inflammation, and identify tiny difference among a large patient population. Although GPS was also found to be independently associated with OS (*p* = 0.001) as well as the CRP/Alb ratio, there were 59.3% of patients classified in the group of GPS 0, which implied that the GPS could not accurately predict the prognosis of a large proportion of our patients. Moreover, the GPS was not statistically different in the OS between the patients with GPS 1 and those with GPS 2. More interestingly, we could enrich the prognostic information of CRP-albumin-based model (aGPS) when we introduced the CRP/Alb ratio into the model along with GPS. Similar findings were also reported by Wei et al. [45], in which the patients with GPS 0 could be categorized into two risk subgroups by the CRP/Alb ratio. Furthermore, aGPS was significantly associated with OS when stratified by plasma EBV-DNA level in our cohort. From this point of view, aGPS which was consisted of three parameters (CRP, albumin, and CRP/Alb ratio) might serve as a more accurate prognostic factor than GPS and provide new insight into the inflammatory prognostic system. However, the prognostic efficacy of aGPS should be further explored in prospective studies and in other cancers other than in NPC.

The association of the CRP/Alb ratio with other clinical characteristics was also analyzed in our study. It was found that the high CRP/Alb ratio subgroup had significantly higher NLR, PLR, and pretreatment EBV-DNA level and lower hemoglobin level compared with the low CRP/Alb ratio subgroup. Based on these findings, it was suggested that the CRP/Alb ratio could not only reflect the individual's systematic inflammatory response but also perform as an indicator of nutritional status.

However, inconsistent with the results of Xu et al. [33] and Wei et al. [45] studies, our data did not show a significant association between GPS and the CRP/Alb ratio. From this point, it was suggested that the combination of GPS and the CRP/Alb ratio might be more informative on inflammation and nutrition than either one alone in NPC patients.

In contrast to localized NPC with curative treatment, the prognostic factors were less identified in metastatic NPC. Plasma EBV-DNA load has been long established as a strong indicator of tumor burden and as a reliable prognostic factor in both disseminated NPC and localized NPC [4, 21, 24]. In line with these data, high pretreatment EBV-DNA level was also found to be an unfavourable prognostic factor in our study. Besides EBV-DNA level, other prognostic factors such as LDH level [21, 22] and NLR [26] have been identified in metastatic NPC. Baseline LDH level, haemoglobin level, number of metastasis site, NLR, and PLR were also analyzed in the current study and showed a close association with the CRP/Alb ratio. However, they failed to independently predict prognosis in the multivariate analysis, indicating that they reflect tumor burden less sensitively and less precisely than the CRP/Alb ratio.

The optimal cutoff value for the CRP/Alb ratio varied in different studies. Even in the two studies of esophageal squamous cell carcinoma (ESCC), the cutoff values were different. In the study by Xu et al. [33], the cutoff was set as 0.50, which was much higher than that in the study by Wei et al. (0.095) [45], due to different methods and different patient population. In our study, we used the same method as Wei et al. [45] and determined the cutoff value as 0.189. In the study investigating the CRP/Alb ratio in nonmetastatic NPC, 0.064 was determined as the optimal cutoff value [36].

Generally speaking, there were three main superiorities in this study. First, we explored the association of the CRP/Alb ratio with treatment response and chemotherapy regimen, which was not always discussed in previous studies. Second, all patients underwent at least two cycles of cisplatin-based chemotherapy, and the heterogeneity of therapeutic options might minimize the potential interference from the variation of different treatments. By contrast, most of previous studies included patients with noncisplatin chemotherapy. Xia et al. [25] enrolled two patients who underwent sorafenib, which was not recommended as standard first-line treatment. In the study by Jin et al. [21], 5% of the patients received monotherapy with capecitabine for their poor performance status. Toh et al. [20] did not describe the detailed treatment for their study cohorts, and 40% of the patients did not undergo any salvage chemotherapy.

Since it could be easily calculated from the routine clinical laboratory tests, testing the CRP/Alb ratio could be used as a simple, feasible, inexpensive method to predict prognosis and to monitor the dynamic change of tumor burden. It might be possible that early management of systematic inflammation and nutritional support could increase the survival of patients with disseminated NPC. Since the nonsteroidal anti-inflammatory drugs (NSAIDs) have showed promising effect on preventing carcinogenesis and reducing the risk of death in various cancers [46–49], NSAIDs might be a feasible and convenient drug to modulate the inflammation of NPC patients. Moreover, whether dynamic change of the CRP/Alb ratio during the treatment course could predict prognosis and treatment response remains unknown. Future prospective clinical trials and deeper basic research could address these questions and provide more reliable molecular and genetic mechanisms regarding the CRP/Alb ratio.

There were several limitations to be acknowledged in this study. First, this was a retrospective study limited to a single institute. Second, another independent cohort was not introduced to validate the novel prognostic index. Finally, we could not provide complete information on disease-free interval (DFI), which was previously established as an important prognostic factor and thus did not further discuss it in our study. All the inferiorities should be overcome by prospective, multicentre studies.

## 5. Conclusion

In summary, our study demonstrated that the CRP/Alb ratio was an independent prognostic factor in metastatic NPC. The CRP and Alb levels could be easily, feasibly, inexpensively examined and therefore it is convenient to use the CRP/Alb ratio in our clinical practice to evaluate systematic inflammation and predicate the survival outcome in metastatic NPC. In addition, we proposed a new prognostic score system based on both the GPS and the CRP/Alb ratio. Prospective multicentre studies are warranted to validate this model and to further explore the underlying mechanisms.

## Figures and Tables

**Table 1:** Baseline clinical features of 148 patients with metastatic nasopharyngeal carcinoma.

Characteristic	Number (%)	CRP/Alb < 0.189, number (%)	CRP/Alb ≥ 0.189, number (%)	*p* value
Gender (male/female)	124/24 (83.8/16.2)	63/15 (80.8/19.2)	61/9 (87.1/12.9)	0.373
Age, years (median/range)	45/24–72	45/26–70	43.5/24–72	0.765
KPS (median/range)	90/60–100	90/60–100	90/60–100	0.276
Number of involved sites (one/multiple)	69/79 (46.6/53.4)	42/36 (53.8/46.2)	27/43 (38.6/61.4)	0.071
Synchronous metastasis (yes/no)	43/105 (29.1/70.9)	20/58 (25.6/74.4)	23/47 (32.9/67.1)	0.368
Liver metastasis (yes/no)	56/92 (37.8/62.2)	28/50 (35.9/64.1)	28/42 (40/60)	0.615
Lung metastasis (yes/no)	69/79 (46.6/53.4)	38/40 (48.7/51.3)	31/39 (44.3/55.7)	0.623
Bone metastasis (yes/no)	61/87 (41.2/58.8)	28/50 (35.9/64.1)	33/37 (47.1/52.9)	0.184
Smoking (yes/no)	68/80 (45.9/54.1)	36/42 (42.1/53.8)	32/38 (45.7/54.3)	1.000
GPS (0/1/2)	88/46/14 (59.5/31.1/9.4)	52/20/6 (66.7/25.6/7.7)	36/26/8 (51.4/37.1/11.4)	0.169
Serum LDH, U/L (median/range)	202.5/25–2975	179.5/122–2821	230/25–2975	0.069
EBV-DNA, copies/mL (median/range)	4.82 × 104/0–9.13 × 107	3.49 × 104/0–9.87 × 106	5.32 × 105/0–9.13 × 107	0.013^∗^
Chemotherapy regimen (TP/PF/TPF)	12/45/91 (8.1/30.4/61.5)	7/26/45 (9/33.3/57.7)	5/19/46 (7.2/27.1/65.7)	0.605
Treatment response (CR + PR/PD + SD)	107/41 (72.3/27.7)	59/19 (75.6/24.4)	48/22 (68.6/31.4)	0.363
NLR (median/range)	3.3/1–35.2	2.8/1–14	4/1–35.2	0.001^∗^
PLR (median/range)	181/56.4–820	161/88–735	231/56.4–820	0.001^∗^
Hemoglobin, g/L (median/range)	131/43–171	132/43–171	123/82–162	0.019^∗^

CRP/Alb: C-reactive protein/albumin ratio; KPS: Karnofsky Performance Score; GPS: Glasgow Prognostic Score; NLR: neutrophil to lymphocyte ratio; PLR: platelet-to-lymphocyte ratio; ^∗^*p* < 0.05.

**Table 2:** Univariate and multivariate analysis of OS in 148 patients with metastatic nasopharyngeal carcinoma.

Variable	Univariate		Multivariate	
	*p* value	HR (95% CI)	*p* value	HR (95% CI)
Gender (male/female)	0.570	0.83 (0.435–1.581)		
Age, years (<50/≥50)	0.773	1.078 (0.646–1.801)		
KPS (≥90/<90)	0.411	0.765 (0.403–1.451)		
Number of involved sites (one/multiple)	0.179	1.369 (0.866–2.164)		
Synchronous metastasis (yes/no)	0.168	0.704 (0.427–1.160)		
Liver metastasis (yes/no)	0.802	0.942 (0.593–1.498)		
Lung metastasis (yes/no)	0.389	1.219 (0.777–1.914)		
Bone metastasis (yes/no)	0.071	1.525 (0.965–2.411)		
Smoking (yes/no)	0.210	0.750 (0.478–1.177)		
GPS (0/1/2)	<0.001^∗^	3.135 (2.309–4.256)	0.001^∗^	2.137 (1.369–3.337)
CRP/Alb (<0.189/≥0.189)	0.003^∗^	1.998 (1.253–3.185)	0.024^∗^	1.867 (1.085–3.210)
Serum LDH, U/L (<212/≥212)	0.006	1.880 (1.193–2.962)	0.602	0.858 (0.483–1.524)
EBV-DNA, copies/mL (<4.82 × 104/≥4.82 × 104)	<0.001^∗^	4.554 (2.792–7.427)	0.041^∗^	2.012 (1.027–3.941)
Chemotherapy regimen (PF/TP/TPF)	0.644	0.922 (0.653–1.302)		
Treatment response (CR + PR/PD + SD)	0.005^∗^	1.984 (1.231–3.197)	0.420	1.242 (0.734–2.101)
NLR (<5/≥5)	0.017^∗^	2.039 (1.133–3.671)	0.192	1.522 (0.810–2.858)
PLR (<152/≥152)	0.002^∗^	2.450 (1.407–4.267)	0.128	1.617 (0.870–3.003)
Hemoglobin, g/L (<11/≥11)	0.585	1.172 (0.664–2.069)		

CRP/Alb: C-reactive protein/albumin ratio; KPS: Karnofsky Performance Score; GPS: Glasgow Prognostic Score; NLR: neutrophil to lymphocyte ratio; PLR: platelet-to-lymphocyte ratio; LDH: lactate dehydrogenase; ^∗^*p* < 0.05.

## Abbreviations

ALP: Alkaline phosphatase
CRP/Alb: The ratio of CRP/albumin
CRP: C-reactive protein
DFI: Disease-free interval
EBV: Epstein-Barr virus
ESCC: Esophageal squamous cell carcinoma
GPS: Glasgow Prognostic Score
HRs: Hazard ratios
IMRT: Intensity modulated radiotherapy technology
KPS: Karnofsky Performance Scores
LDH: Lactate dehydrogenase
mGPS: Modified Glasgow Prognostic Score
MLR: Monocyte to lymphocyte ratio
NLR: Neutropil to lymphocyte ratio
NPC: Nasopharyngeal carcinoma
NSAIDs: Nonsteroidal anti-inflammatory drugs
PF: Cisplatin plus 5-fluorouracil
PLR: Platelet-to-lymphocyte ratio
PS: Performance status
RECIST: Response Evaluation Criteria in Solid Tumors
TNM: Tumor-node-metastasis
TP: Paclitaxel plus cisplatin
TPF: Paclitaxel plus cisplatin plus 5-fluorouracil.
